# Position and Velocity Estimation for Two-Inertia System with Nonlinear Stiffness Based on Acceleration Sensor

**DOI:** 10.3390/s16010049

**Published:** 2015-12-31

**Authors:** Kyung-Tae Nam, Seung-Joon Lee, Tae-Yong Kuc, Hyungjong Kim

**Affiliations:** 1 Department of Electronic Engineering, Sungkyunkwan Univerity, Suwon 440-746, Korea; robotnam@kitech.re.kr (K.-T.N.); tykuc@skku.edu (T.-Y.K.); 2 Korea Institute of Industrial Technology, Ansan 426-171, Korea; sjlee10@kitech.re.kr; 3 ASRI, Department of Electrical and Computer Engineering, Seoul National University, Seoul 151-744, Korea

**Keywords:** flexible joint manipulators, state estimation, acceleration, nonlinear stiffness, Lipschitz constant, FPD transfer robot

## Abstract

In this paper, we consider the state estimation problem for flexible joint manipulators that involve nonlinear characteristics in their stiffness. The two key ideas of our design are that (a) an accelerometer is used in order that the estimation error dynamics do not depend on nonlinearities at the link part of the manipulators and (b) the model of the nonlinear stiffness is indeed a Lipschitz function. Based on the measured acceleration, we propose a nonlinear observer under the Lipschitz condition of the nonlinear stiffness. In addition, in order to effectively compensate for the estimation error, the gain of the proposed observer is chosen from the ARE (algebraic Riccati equations) which depend on the Lipschitz constant. Comparative experimental results verify the effectiveness of the proposed method.

## 1. Introduction

Flexible joint manipulators are widely used in industrial applications that require high productivity [1,2]. Furthermore, they can deal with many kinds of assembling, manufacturing, and moving jobs with low costs. Thus, for several decades, a lot of effective control methods have been proposed [1,2,3,4,5,6,7,8]. While the majority of the proposed controllers require exact state information, such as the position and the velocity of the motor and the link, it is not easy to obtain this exact state information due to the high nonlinearity, high coupling, and model uncertainty. In particular, the estimation of link states is important because most industrial manipulators are not usually equipped with sensors. To this end, research has been conducted [4,9,10]. However, it is still difficult to obtain link information because the motor position is only measurable and the manipulators demonstrate flexibility between the motor and link.

**Figure 1:**
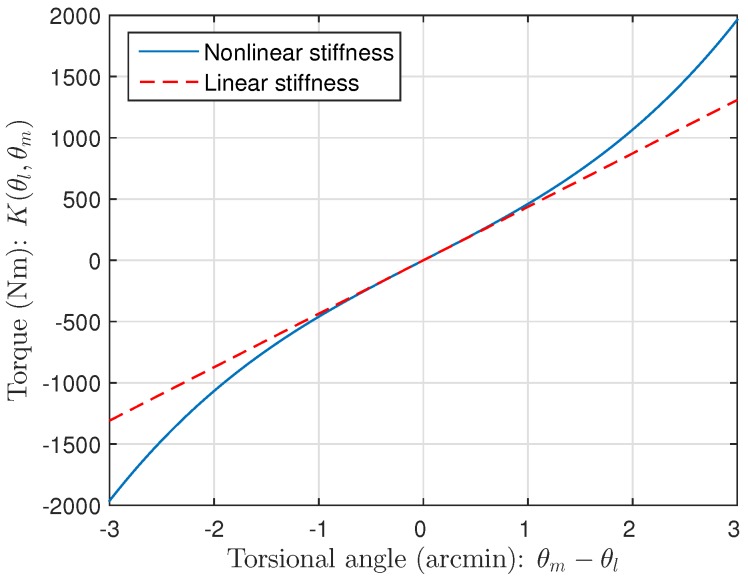
Nonlinear stiffness.

Recently, observers based on the acceleration information have been proposed to obtain more accurate states [11,12,13,14,15]. By the accelerometer which is mounted on the link of the robot manipulator, the observer uses information of link acceleration, and thus the complexity of the link part can be eliminated. As a result, the estimation error can be made globally asymptotically stable for flexible joint manipulators with linear stiffness. However, as shown in Figure 1, the flexible joint manipulators actually have nonlinear characteristics in stiffness that appear when the torsional angle between the motor and the link increases [16,17,18].

In this paper, we propose an acceleration based nonlinear observer that takes into consideration the nonlinear stiffness of the robot manipulator. We transform the robot model with nonlinear stiffness into a Lipschitz nonlinear system [19], and then design the observer of the transformed system. The observer gain is designed by the ARE (algebraic Riccati equations) in order that the observer error asymptotically converges to zero.

The paper adheres to the following organizational structure: In Section 2, we present the problem formulation. Section 3 provides an observer design method and stability analysis. Then, in Section 4, the proposed observer is experimentally tested in terms of real industrial robots. Finally, conclusions are laid out Section 5.

## 2. Problem Formulation

While the eventual goal of this paper is to estimate the states of the multiaxis flexible joint manipulator, we consider the observation problem for a two-inertia system since it appropriately describes the dynamic characteristics of a single manipulator with a flexible joint [20]. The two-inertia system is described by [16]

(1) $$\begin{array}{c} {\dot{\theta }}_{l}=\omega_{l}\\ {\dot{\omega }}_{l}=J_{l}^{-1}\left(\theta_{l}\right)\left(D\left(\omega_{m}-\omega_{l}\right)+K\left(\theta_{l},\theta_{m}\right)-C\left(\theta_{l},\omega_{l}\right)-G\left(\theta_{l}\right)\right)\\ {\dot{\theta }}_{m}=\omega_{m}\\ {\dot{\omega }}_{m}=J_{m}^{-1}\left(D\left(\omega_{l}-\omega_{m}\right)-K\left(\theta_{l},\theta_{m}\right)\right)+J_{m}^{-1}\tau \end{array}$$

where $\theta_{l}$ and $\theta_{m}$ are the angular positions of the link and motor, and $\omega_{l}$ and $\omega_{m}$ are the angular velocities of the link and motor, respectively. The signal *τ* is the torque applied to the motor. The link inertia $J_{l}\left(\theta_{l}\right)$ and the gravity term $G(\theta_{l})$ depend on the position of the link, and Coriolis and centrifugal term $C(\theta_{l},\omega_{l})$ depend on the position and angular velocity of the link, while the motor inertia $J_{m}$ and damping *D* have constant values. The nonlinear stiffness function $K(\theta_{l},\theta_{m})$ is given by

(2) $$\begin{array}{c} K\left(\theta_{l},\theta_{m}\right)=\left\{\begin{array}{cc} -k_{1}\theta_{B}-k_{2}\theta_{B}^{3}-\left(k_{1}+3k_{2}\theta_{B}^{2}\right)\left(-\theta_{m}+\theta_{l}-\theta_{B}\right), & \text{if}\;\theta_{m}-\theta_{l}<-\theta_{B}\\ k_{1}\left(\theta_{m}-\theta_{l}\right)+k_{2}{\left(\theta_{m}-\theta_{l}\right)}^{3}, & \text{if}\;∥\theta_{m}-\theta_{l}∥\leq \theta_{B}\\ k_{1}\theta_{B}+k_{2}\theta_{B}^{3}+\left(k_{1}+3k_{2}\theta_{B}^{2}\right)\left(\theta_{m}-\theta_{l}-\theta_{B}\right), & \text{if}\;\theta_{m}-\theta_{l}>\theta_{B} \end{array}\right. \end{array}$$

where the positive numbers $k_{1}$ and $k_{2}$ represent the linear and nonlinear coefficients of spring stiffness, respectively. The breakpoint deflection $\theta_{B}$ is a positive constant, which refers to the physical limit of the torsional angle between the motor and link.

Our goal is to design an observer that guarantees the estimation performance of all the states of the Equation (1). In particular, it is important to estimate the states of the link part because of the lack of the available position sensors on the link side.

Now, some assumptions are made, on which the proposed observer will be designed in the next section.

**Assumption 1.** *The motor position $\theta_{m}$ and the link acceleration ${\dot{\omega }}_{l}$ are measurable while $\theta_{l}$, $\omega_{l}$, and $\omega_{m}$ are not. *◊

**Assumption 2.** *The system parameters $J_{m},D,k_{1},k_{2}$, and $\theta_{B}$ are known. *◊

## 3. Main Results

### 3.1. Observer Design

Define $\theta_{d}:=\theta_{m}-\theta_{l}$. Then, the Equation (2) is divided into a linear part and a nonlinear part of $\theta_{d}$, and thus it follows from Equations (1) and (2) that

(3) $$\begin{array}{c} \begin{array}{cc} \dot{x} & =Ax+Y+\Phi (x)+Bu\\ y_{1} & =Cx \end{array} \end{array}$$

where u:=τ is the input, $x:={\left[\begin{array}{cccc} x_{1} & x_{2} & x_{3} & x_{4} \end{array}\right]}^{T}:={\left[\begin{array}{cccc} \theta_{l} & \omega_{l} & \theta_{d} & \omega_{m} \end{array}\right]}^{T}$ are the states, $y:={\left[\begin{array}{cc} y_{1} & y_{2} \end{array}\right]}^{T}:={\left[\begin{array}{cc} \theta_{m} & {\dot{\omega }}_{l} \end{array}\right]}^{T}$ are the measurable outputs, and

$$\begin{array}{cc} A & =\left[\begin{array}{cccc} 0 & 1 & 0 & 0\\ 0 & 0 & 0 & 0\\ 0 & -1 & 0 & 1\\ 0 & J_{m}^{-1}D & -J_{m}^{-1}k_{1} & -J_{m}^{-1}D \end{array}\right],\;Y=\left[\begin{array}{c} 0\\ y_{2}\\ 0\\ 0 \end{array}\right]\\ \Phi (x) & =\left[\begin{array}{c} 0\\ 0\\ 0\\ -J_{m}^{-1}\phi \left(x_{3}\right) \end{array}\right],\;B=\left[\begin{array}{c} 0\\ 0\\ 0\\ J_{m}^{-1} \end{array}\right],\;C={\left[\begin{array}{c} 1\\ 0\\ 1\\ 0 \end{array}\right]}^{T}\\ \phi (x_{3}) & =\left\{\begin{array}{cc} \phi_{1}\left(x_{3}\right)=3k_{2}\theta_{B}^{2}x_{3}+2k_{2}\theta_{B}^{3}, & \text{if}\;x_{3}<-\theta_{B}\\ \phi_{2}\left(x_{3}\right)=k_{2}x_{3}^{3}, & \text{if}\;∥x_{3}∥\leq \theta_{B}\\ \phi_{3}\left(x_{3}\right)=3k_{2}\theta_{B}^{2}x_{3}-2k_{2}\theta_{B}^{3}, & \text{if}\;x_{3}>\theta_{B} \end{array}\right. \end{array}$$

Note that the matrix Φ(x) is a nonlinear function of $\phi (x_{3})$, and thus we obtain the following.

**Lemma 1.**  *The function Φ(x) is globally Lipschitz, i.e., there exists a Lipschitz constant $\gamma (=J_{m}^{-1}3k_{2}\theta_{B}^{2})>0$ such that the following property holds.*

$$\begin{array}{c} ∥\Phi \left(x\right)-\Phi \left(\hat{x}\right)∥\leq \gamma ∥x-\hat{x}∥,{\;}^{\forall }x,\hat{x}\in R^{4} \end{array}$$

**◊

Now, we propose a nonlinear observer for the System (3) as follows:

(4) $$\begin{array}{c} \begin{array}{cc} \dot{\hat{x}} & =A\hat{x}+Y+\Phi \left(\hat{x}\right)+Bu+L\left(y_{1}-{\hat{y}}_{1}\right)\\ {\hat{y}}_{1} & =C\hat{x} \end{array} \end{array}$$

where *L* is a suitable observer gain (which will be designed in the following).

Define the estimation error by $e:=x-\hat{x}$. Then, the estimation error dynamic is seen to be given by

(5) $$\begin{array}{cc} \dot{e} & =\left(A-LC\right)e+\left(\Phi \left(x\right)-\Phi \left(\hat{x}\right)\right)\\ & =:A_{ob}e+\left(\Phi \left(x\right)-\Phi \left(\hat{x}\right)\right) \end{array}$$

In order to stabilize the error System (5), it is of great importance to design an appropriate observer gain *L*. When the nonlinear term $\left(\Phi \left(x\right)-\Phi \left(\hat{x}\right)\right)$ is zero, the stability of error dynamics is guaranteed if the observer gain *L* is designed such that the matrix A-LC is Hurwitz (*i.e.*, all its eigenvalues have negative real parts). However, since the Equation (5) has a nonlinear term, the Lipschitz function, we have to use a different method. There are some results on the study that consider the stability of Estimation Error Dynamics (5) [19,21]. We briefly introduce a result in [19] to design the observer gain matrix *L*. For some small ϵ>0, if the following the ARE (algebraic Riccati equation)

(6) $$\begin{array}{c} AP+PA^{T}+P\left(\gamma^{2}I-\frac{1}{\epsilon }C^{T}C\right)P+I+\epsilon I=0 \end{array}$$

has a symmetric positive definite solution *P*. Then, the observer gain

(7) $$\begin{array}{c} L=\frac{PC^{T}}{2\epsilon } \end{array}$$

stabilizes the Estimation Error Eynamics (5).

We shall now proceed to state the main results of this paper.

**Theorem 1.**  *Suppose the observer is given by Equation (4). Then, under Assumptions 1 and 2, the Estimation Error Dynamic (5) is asymptotically stable if the algebraic Ricaati Equation (6) has a symmetric positive definite solution P and the observer gain is designed by Equation (7). *◊

**Proof.**  From the Equations (6) and (7), we obtain

(8) $$\begin{array}{c} A_{ob}P+PA_{ob}^{T}+\gamma^{2}PP+I<0 \end{array}$$

Then, by [19] (Lemma 1), we have

(9) $$\begin{array}{c} A_{ob}^{T}P_{1}+P_{1}A_{ob}+\gamma^{2}P_{1}P_{1}+I<0 \end{array}$$

where $P_{1}$ is any symmetric positive definite matrix. Consider the Lyapunov function candidate

$$V=e^{T}P_{1}e$$

By Lemma 1, its derivative is given by

(10) $$\begin{array}{cc} \dot{V} & =e^{T}\left(A_{ob}^{T}P_{1}+P_{1}A_{ob}\right)e+2e^{T}P_{1}\left(\Phi \left(x\right)-\Phi \left(\hat{x}\right)\right)\\ & \leq e^{T}\left(A_{ob}^{T}P_{1}+P_{1}A_{ob}\right)e+2∥P_{1}e∥∥\left(\Phi \left(x\right)-\Phi \left(\hat{x}\right)\right)∥\\ & \leq e^{T}\left(A_{ob}^{T}P_{1}+P_{1}A_{ob}\right)e+2\gamma ∥P_{1}e∥∥e∥\\ & \leq e^{T}\left(A_{ob}^{T}P_{1}+P_{1}A_{ob}\right)e+\gamma^{2}e^{T}P_{1}P_{1}e+e^{T}e\\ & =e^{T}\left(A_{ob}^{T}P_{1}+P_{1}A_{ob}+\gamma^{2}P_{1}P_{1}+I\right)e \end{array}$$

It follows from the Equation (9) that $\dot{V}<0$, and so the Estimation Error Dynamics (5) is asymptotically stable by [22] (Theorem 4.1). ☐

**Remark 1.** *Instead of the results from [19], we can consider the high gain observer proposed in [23] because the proposed Observer (4) does not guarantee the solution of the ARE exists. In fact, the observer gain of [23] does not require the resolution of any equation and is explicitly given. However, the System (3) does not satisfy the necessary assumptions of [23] because of the nonlinear term Φ(x). *◊

### 3.2. Coordinate Transformation

Since the manipulator systems in industrial fields usually have large coefficients of spring stiffness function, the magnitude of *γ* also has a large value. If *γ* is too large to satisfy the conditions in which the ARE (6) has a symmetric positive definite solution, then we cannot find the positive definite solution *P* satisfying the ARE since the real values of the eigenvalues of the Hamiltonian matrix for the ARE are close to zero [24]. Thus, in order to reduce the Lipschitz constant, we use the coordinate transformation method proposed in [21].

Let us define a transformation matrix

(11) $$\begin{array}{c} T:=\left[\begin{array}{cccc} 1 & 0 & 0 & 0\\ 0 & 1 & 0 & 0\\ 0 & 0 & 1 & 0\\ 0 & 0 & 0 & \beta \end{array}\right] \end{array}$$

where *β* is any small positive number. Suppose z:=Tx, then, the System (3) becomes

(12) $$\begin{array}{c} \begin{array}{cc} \dot{z} & =TAT^{-1}z+TY+T\Phi \left(T^{-1}z\right)+TBu\\ y_{1} & =CT^{-1}z \end{array} \end{array}$$

where $z_{1}=x_{1}$, $z_{2}=x_{2}$, $z_{3}=x_{3}$, $z_{4}=\beta x_{4}$, and $T\Phi \left(T^{-1}z\right)={\left[\begin{array}{cccc} 0 & 0 & 0 & -\beta J_{m}^{-1}\phi \left(z_{3}\right) \end{array}\right]}^{T}$. Similarly, with $\hat{z}:=T\hat{x}$, the Equation (4) becomes

(13) $$\begin{array}{c} \begin{array}{cc} \dot{\hat{x}} & =TAT^{-1}\hat{z}+TY+T\Phi \left(T^{-1}\hat{z}\right)+TBu+TL\left(y_{1}-{\hat{y}}_{1}\right)\\ {\hat{y}}_{1} & =CT^{-1}\hat{z} \end{array} \end{array}$$

Then, with $e_{z}:=z-\hat{z}$, the estimation error dynamics in the new coordinate are seen to be given by:

(14) $$\begin{array}{cc} {\dot{e}}_{z} & =T\left(A-LC\right)T^{-1}e_{z}+T\left(\Phi \left(T^{-1}z\right)-\Phi \left(T^{-1}\hat{z}\right)\right)\\ & =:{\tilde{A}}_{ob}e_{z}+T\left(\Phi \left(T^{-1}z\right)-\Phi \left(T^{-1}\hat{z}\right)\right) \end{array}$$

Here, the Lipschitz constant *γ* in Lemma 1 is changed by the transformation matrix *T* as follows:

(15) $$\begin{array}{cc} & ∥T\Phi \left(T^{-1}z\right)-T\Phi \left(T^{-1}\hat{z}\right)∥\\ & =∥\left[\begin{array}{c} 0\\ 0\\ 0\\ -\beta J_{m}^{-1}\phi \left(z_{3}\right) \end{array}\right]-\left[\begin{array}{c} 0\\ 0\\ 0\\ -\beta J_{m}^{-1}\phi \left({\hat{z}}_{3}\right) \end{array}\right]∥\\ & \leq \beta J_{m}^{-1}∥\phi \left(z_{3}\right)-\phi \left({\hat{z}}_{3}\right)∥\\ & \leq \beta \gamma ∥z_{3}-{\hat{z}}_{3}∥∥=:\tilde{\gamma }∥z_{3}-{\hat{z}}_{3}∥ \end{array}$$

Then, similar to the Equation (6), we obtain the following the ARE with the new Lipschitz constant $\tilde{\gamma }=\beta \gamma$

(16) $$\begin{array}{c} \tilde{A}\tilde{P}+\tilde{P}{\tilde{A}}^{T}+\tilde{P}\left({\tilde{\gamma }}^{2}I-\frac{1}{\epsilon }{\tilde{C}}^{T}\tilde{C}\right)\tilde{P}+I+\epsilon I=0 \end{array}$$

where $\tilde{A}:=TAT^{-1}$ and $\tilde{C}:=CT^{-1}$. Therefore, if the ARE (16) has a symmetric positive definite solution $\tilde{P}$, then the new observer gain

(17) $$\begin{array}{c} L=T^{-1}\frac{\tilde{P}{\tilde{C}}^{2}}{2\epsilon } \end{array}$$

stabilizes the Estimation Error Dynamics (5), and also stabilizes the System (5) by $e=T^{-1}e_{z}$.

## 4. Experimental Results

**Figure 2:**
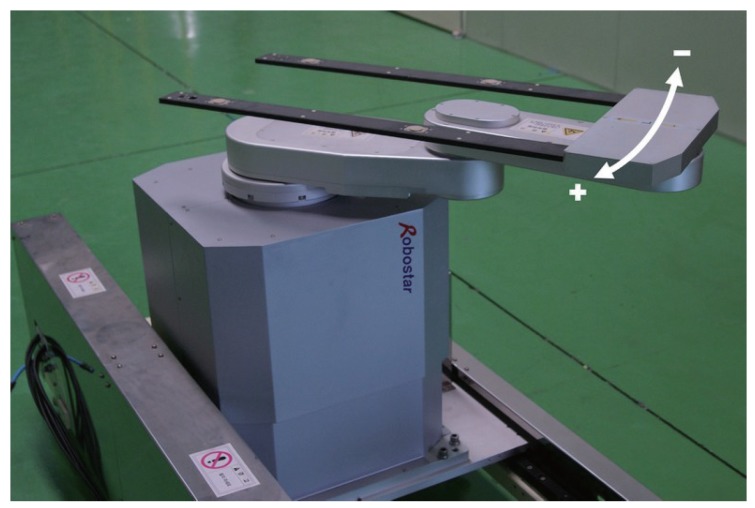
FPD (flat panel display) transfer robot.

In this section, an experiment on a real industrial robot was carried out in order to verify the effectiveness of the observer proposed in this paper. As shown in Figure 2, the FPD (flat panel display) transfer robot is used in this experiment. As a matter of fact, most of the industrial manipulators just have an encoder in the motor part and not the link part, but the FPD robot also has an encoder in order to measure the link position. Consequently, without any position measurement system, we can compare the actual link position and the estimated value from the designed observer. This is why we used the FPD robot instead of the typical six-joint manipulator shown in [1,2,15]. The parameters of this robot system are given in Table 1. To obtain the acceleration information of the link side, we also use *Miniature DeltaTron Accelerometer Type 4508B* (manufactured by Brüel & Kjr). The design parameters for the proposed Observer (4) are selected as shown Table 2. The observers are implemented through Matlab xPC Target and the sampling rate is 2 kHz.

**Table 1:** System parameters.

Parameter	Value	Unit
motor inertia ($J_{m}$)	0.001027	kg· m${}^{2}$
damping (*D*)	600	Nm·s/rad
$k_{1}$	1.5 $\times 10^{6}$	Nm/rad
$k_{2}$	9.85 $\times 10^{11}$	Nm/rad${}^{3}$
$\theta_{B}$	2	arcmin
gear ratio	144	

**Table 2:** Design parameters.

Parameter	Value
observer gain (*L*)	${\left[-15968,\;-8506,\;19896,\;154\right]}^{T}$
*ϵ*	$1.0\times 10^{-6}$
*β*	$1.0\times 10^{-5}$

**Figure 3:**
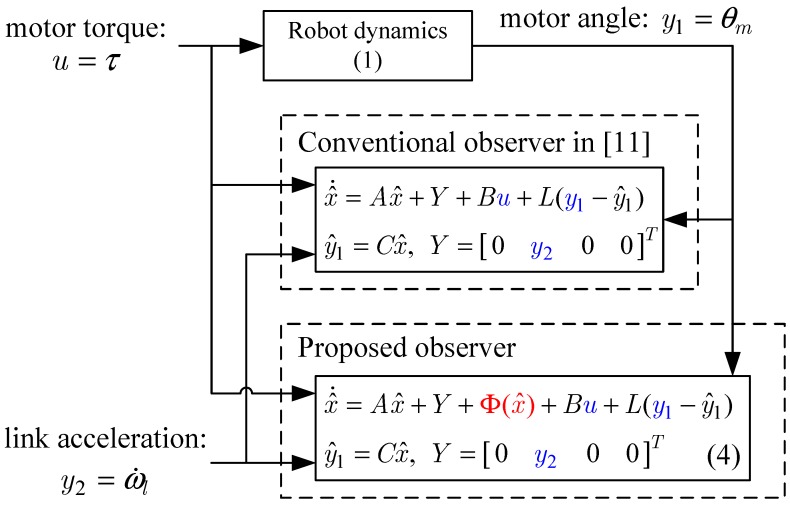
Block diagram of the robot system with the conventional and proposed observer.

**Figure 4:**
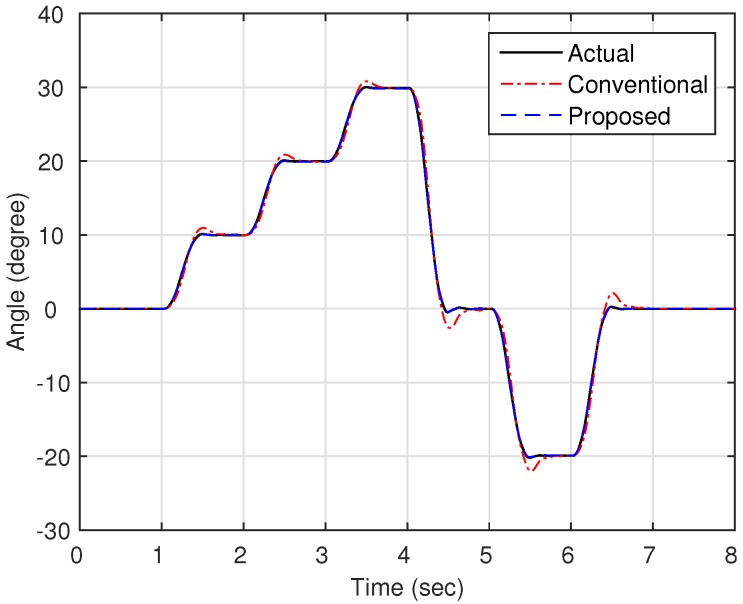
Trajectory of the link position $\theta_{l}$.

**Figure 5:**
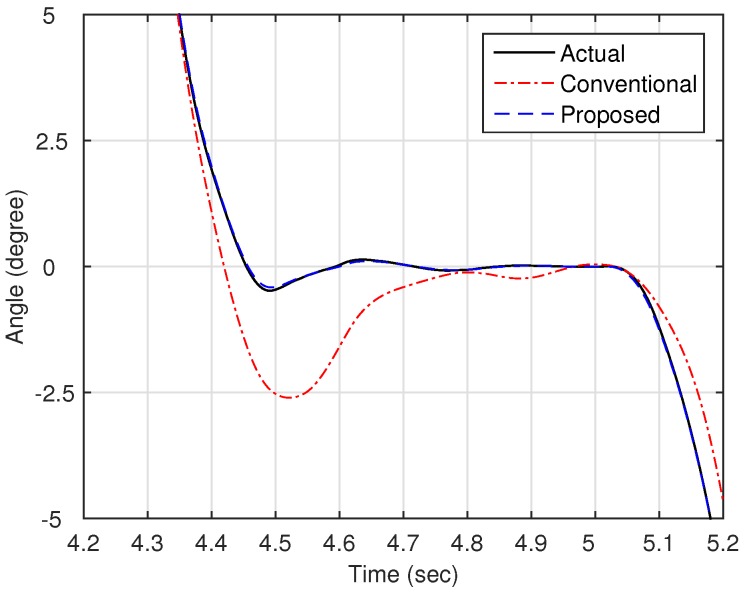
Magnified trajectory of the link position (around 4.7 s).

**Figure 6:**
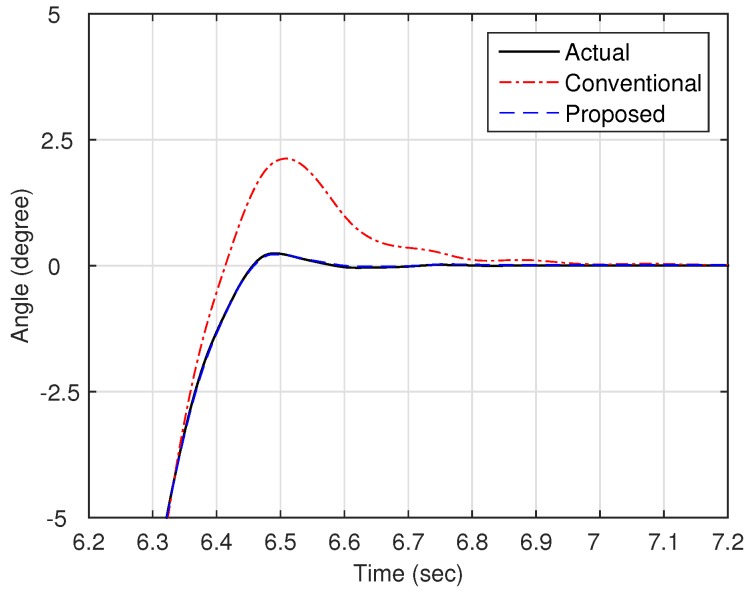
Magnified trajectory of the link position (around 6.7 s).

**Figure 7:**
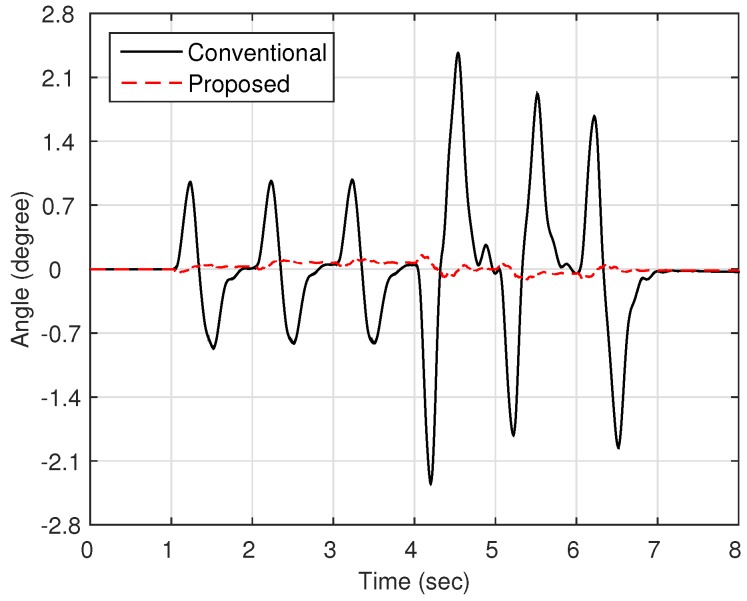
Estimation error of the link position $\theta_{l}$.

**Figure 8:**
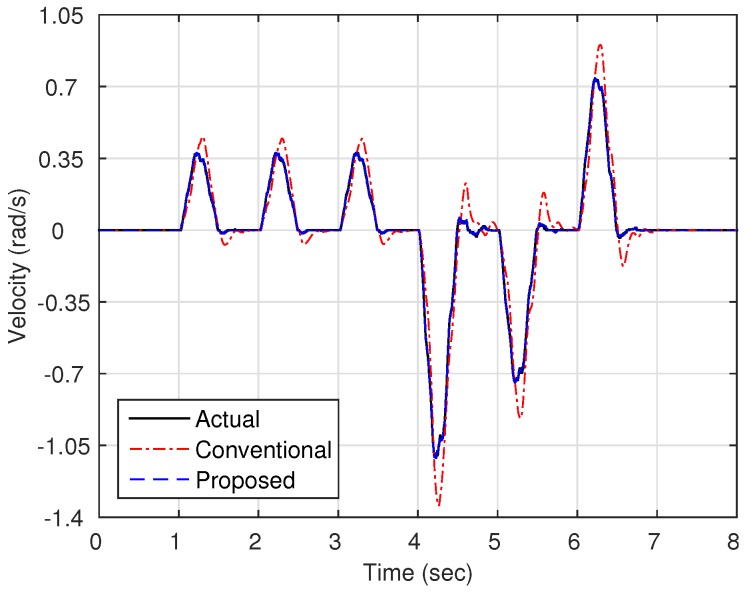
Trajectory of the link velocity $\omega_{l}$.

Now, as shown in Figure 3, we compare the estimation performances of the proposed observer and the observer with the linear stiffness proposed in [11]. Specifically, we force on the estimation performance of the link states (position and velocity) since the motor states are typically obtained from the sensors such as encoders. Figure 4 shows the trajectory of the link position for cases of multi motion. The positive angle means that the robot arm rotates in a clockwise direction, whereas the negative angle implies a counter-clockwise direction. The black solid line is the measured value from the encoder, and the red dash-dot line and the blue dashed line are the estimated value of the conventional observer and proposed observer, respectively. In order to examine the performance in more detail, we magnify Figure 4 at 4.7 s and 6.7 s, respectively, as shown in Figure 5 and Figure 6. In addition, Figure 7 shows the estimation error of the link position of Figure 4. We note that the estimation performance of proposed observer is better than the conventional observer. In particular, it is observed that the estimation error is better suppressed with the proposed observer in transition response because the torsional angle increased in transition is the cause of the characteristic of the nonlinear stiffness as shown in Figure 1. On the other hand, the characteristic of nonlinear stiffness weakens in the steady-state since the torsional angle approaches the origin. Similarly, as shown in Figure 8, the estimation performance of the link velocity is also better than the conventional observer.

## 5. Conclusions

In this paper, we have presented a state observer for flexible joint manipulators using the acceleration information of the link side. The observer has been designed on the basis of the Lipschitz nonlinear system, and the stability and performance have been analyzed. In particular, unlike conventional approaches, the study has dealt with the nonlinear stiffness in order to estimate real systems more closely. Therefore, the proposed observer has improved performance compared to that of the conventional observer considering the linear stiffness. Finally, the proposed observer is applied to the real industrial robot, and its effectiveness is confirmed via experiments.

## References

[1] Spong M.W., Hutchinson S., Vidyasagar M. (2006). Robot Modeling and Control.

[2] Bang J.S., Shim H., Park S.K., Seo J.H. (2010). Robust tracking and vibration suppression for a two-inertia system by combining backstepping approach with disturbance observer. IEEE Trans. Ind. Electron..

[3] Tomei P. (1991). A simple pd controller for robots with elastic joints. IEEE Trans. Autom. Control.

[4] Jankovic M. (1995). Observer based control for elastic joint robots. IEEE Trans. Robot. Autom..

[5] Choi C.H., Kwak N. (2003). Robust control of robot manipulator by model-based disturbance attennuation. IEEE ASME Trans. Mechatron..

[6] Islam S., Liu P.X. (2011). Pd output feedback control design for industrial robotic manipulators. IEEE ASME Trans. Mechatron..

[7] Kostarigka A.K., Doulgeri Z., Rovithakis G.A. (2013). Prescribed performance tracking for flexible joint robots with unknown dynamics and variable elasticity. Automatica.

[8] Yun J.N., Su J.B. (2014). Design of a disturbance ovserver for a tow-link manipulator with flexible joints. IEEE Trans. Control Syst. Technol..

[9] Lotfi N., Namvar M. (2010). Global adaptive estimation of joint velocities in robotic manipulators. IET Control Theory Appl..

[10] Cantelli L., Muscato G., Nunnari M., Spina D. (2015). A joint-angle estimation method for industrial manipulators using inertial sensors. IEEE ASME Trans. Mechatron..

[11] Luca A.E., Schroder D., Thummel M., Spina D. An acceleration-based state observer for robot manipulators with elastic joints. Proceedings of the IEEE International Conference on Robotics and Automation.

[12] Staufer P., Gattringer H. (2012). State estimation on flexible robots using accelerometers and angular rate sensors. Mechatronics.

[13] Axelsson P., Karlssn R., Norrlof M. (2012). Bayesian state estimation of a flexible industrial robot. Mechatronics.

[14] Chen W., Tomizuka M. Load side state estimation in robot with joint elasticity. Proceedings of the IEEE/ASME International Conference on Advanced Intelligent Mechatronics (AIM).

[15] Chen W., Tomizuka M. (2014). Direct joint space state estimation in robots with multiple elastic joints. IEEE ASME Trans. Mechatron..

[16] Moberg S., Öhr J., Gunnarsson S. A benchmark problem for robust control of a multivariable nonlinear flexible manipulator. Proceedings of the 17th IFAC World Congress.

[17] Moberg S., Öhr J., Gunnarsson S. (2009). A benchmark problem for robust control of a multivariable nonlinear flexible manipulator. IEEE Trans. Control Syst. Technol..

[18] Moberg S., Wernholt E., Hanssen S., Brongardh T. (2014). Modeling and parameter estimation of robot manipulators using extended flexible joint models. J. Dyn. Syst. Meas. Control.

[19] Raghavan S., Hedrick J.K. (1994). Observer design for a class of nonlinear systems. Asian J. Control.

[20] Sugiura K., Hori Y. (1996). Vibration suppression in 2- and 3-mass system based on the feedback of imperfect derivative of the estimated torsional torque. IEEE Trans. Ind. Electron..

[21] Song B., Hedrick J.K. Nonlinear observer design for lipschitz nonlinear systems. Proceedings of the American Control Conference (ACC).

[22] Khalil H. (2001). Nonlinear Systems.

[23] Farza M., M’Saad M., Rossignol L. (2004). Observer design for a class of MIMO nonlinear systems. Automatica.

[24] Ni M.-L. (2008). Existence condition on solutions to the algebraic riccati equation. Acta Autom. Sin..

